# Soybean oil and leucine in late gestation and lactation improve maternal and offspring lipid metabolism and insulin sensitivity: insights from pig model

**DOI:** 10.3389/fvets.2026.1793347

**Published:** 2026-04-10

**Authors:** Yutong Sun, Zexuan Gan, Xueyuan Li, Yuxin Lin, Qingquan Ma, Xinbo Zhou

**Affiliations:** College of Animal Science and Technology, Northeast Agricultural University, Harbin, China

**Keywords:** insulin sensitivity, leucine, lipid metabolism, offspring, pig, soybean oil

## Abstract

**Introduction:**

Lipid metabolism is crucial for the development of insulin resistance. Leucine supplementation enhances insulin sensitivity and lipid metabolism. Soybean oil has the potential to improve insulin sensitivity. A comprehensive investigation of the effects and potential mechanisms of leucine and soybean oil supplementation is warranted. This study aims to investigate the effects and underlying mechanisms of maternal soybean oil and leucine intake on lipid metabolism, insulin sensitivity in both dams and offspring.

**Materials and methods:**

Sixty-eight multiparous sows (parities 3 to 5, 228.44 ± 8.19 kg) were randomly divided into four groups from the 107th day of gestation to the 21st day of lactation (*n* = 17). The experimental was designed as a 2 × 2 factorial arrangement, which included the level of soybean oil (crude fat 5% or 10%) and leucine (0 or 1%) in the diet.

**Results:**

Supplementing soybean oil during late pregnancy and lactation significantly enhanced insulin sensitivity (*P* < 0.05). Both soybean oil and leucine altered the intestinal microbiota of sows, and significantly improved plasma lipid metabolism (*P* < 0.05). Additionally, inflammation in sows before and after farrowing was reduced by lowering the relative abundance of pro-inflammatory bacterial genus such as *Catenisphaera, Terrisporobacte* and *Peptococcus* (*P* < 0.05). The weaning weight of piglets was significantly increased (*P* < 0.05), along with enhanced insulin sensitivity, increased immune (*IL10RA, DDX58*), and improved lipid metabolism (*PPARA, SLC27A2*) and rhythmic gene expression (*LGR4, KLF9*).

**Discussion:**

Our study provides evidence that dietary supplementation with soybean oil and leucine affects offspring growth by modulating lipid metabolism and insulin sensitivity in sows and pigs, and by altering intestinal microbiota.

## 1 Introduction

Compared with other non-primate models, humans and pigs have similarities in digestive physiology and related metabolic processes (1). Therefore, pigs are often used as model organisms that analyze various human diseases and physiological functions (2).

Metabolic abnormalities and insulin resistance frequently occur in sows from late gestation to lactation (3). The metabolic syndrome is characterized by protein, glucose, lipid, and carbohydrate disorders (4, 5). Research highlights that lipid metabolism and intestinal microbiota balance in sows during the peripartum period are crucial for the development of piglets (1, 6–8). Thus, improving the efficiency of pig production requires alleviating the metabolic burden on sows while retaining their reproductive performance (9). This indicates that it is necessary to find effective functional nutrients and feed ingredients.

Dietary fat is an essential nutrient that in animal diets. Studies indicates that supplementing the diet with fat in late pregnancy and/or during lactation can increase overall litter gain and weaning weight in piglets while reduce body weight loss in sows during lactation (10, 11). A meta-analysis also showed that strategic use of fat during late pregnancy and lactation can enhance sow reproductive performance and litter growth performance (12). Soybean oil is rich in n-6 polyunsaturated fatty acids (PUFAs), has been shown to positively impact lipids, insulin sensitivity, mitochondrial function, and immunity (13–15). Adding 3% soybean oil to the diet of sows during lactation significantly reduces backfat loss (16), backfat thickness management helps regulate maternal and piglet health (17). Although high levels of oil are considered pro-inflammatory to some extent (18).

As nutritional signals, branched-chain amino acids (BCAAs) influence energy and metabolic balance, for instance, feeding behavior and insulin sensitivity (19). Leucine, in particular, regulates glucose and lipid metabolism. The study by Sun et al. also confirmed that leucine can alleviate the effects of early weaning on lipid metabolism and inflammation in piglets (20). The combined administration of high-dose PUFAs and β-hydroxy-β-methylbutyrate (HMB, a key leucine metabolite) demonstrates therapeutic potential for improving metabolic function and cognitive health in clinically stable populations with chronic diseases (21).

Given the potential joint effects of leucine and soybean oil on lipid metabolism and insulin sensitivity, we hypothesized that maternal supplementation with soybean oil and leucine during late gestation and lactation could improve insulin sensitivity by modulating lipid metabolism and enhance piglet growth by altering and gut microbiota.

In this study, we evaluated the beneficial effects of maternal supplementation with soybean oil and leucine during late gestation and lactation on lipid metabolism, insulin sensitivity, and intestinal microbiota in sows and further provided insights into how this supplementation affects piglet growth performance, lipid metabolism, inflammation, and circadian rhythm.

## 2 Materials and methods

### 2.1 Experimental design and animals

This experiment was conducted at DBN Group Swine Breeding CO. LTD. (Daqing, Heilongjiang Province, China). A total of 68 pregnant sows (Large White × Landrace, parity 3–5) were randomly divided into 4 treatments based on body weight at 107 days of gestation and were fed four diets separately, with the details as follows: C (corn-soybean meal diet, crude fat 5%), CL (C+1% leucine), HSO(corn-soybean meal diet, crude fat 10%) and HSOL (HSO+1% leucine). Leucine was purchased from Huayang Biotechnology Co., Ltd., China, and Soybean oil was purchased from Jiusan Grain and Oil Industry Group Company Limited (Harbin, China). The trial lasted from 107 days of gestation to 21 days of lactation. On day 107 of gestation, sows were transferred to a unit equipped with an environmental control system and housed in individual farrowing crates (2.0 × 1.5 m). The sow room was maintained at a temperature of 22 ± 1 °C and with a relative humidity of 70 ± 5%. This experiment used a corn-soybean based diet (Supplementary Table S1) and calculated the diet nutrients to meet the NRC 2012 nutritional recommendations for sows. On the day of farrowing (day 0), the same treatment was cross-fostered and adjusted to 12 ± 1 piglets, the weight of the suckling piglets was observed. Sow milk was the only food source for suckling piglets throughout the experiment. Differences in dietary crude fat (5% and 10%) result from different quantities of added soybean oil. The level of leucine addition was based on previous research findings (20, 22, 23).

### 2.2 Oral glucose tolerance test (OGTT)

Six sows were randomly selected from each treatment for the test, which was conducted on gestational day 107 and lactation day 21. After fasting for 8 h, blood was collected from the caudal vein, and blood glucose levels were measured using an automatic glucose analyzer (Roche Accu-Chek guide Meter, Rotkreuz, Switzerland), which was recorded as 0 min. Then, each sow was administered orally given 0.5g/kg of pure glucose according to body weight. Blood glucose of sows was measured at 15, 30, 60, 90 and 120 min.

### 2.3 Measurement of productive and growth performance

Record the reproductive performance of sows. Use a veterinary digital B-ultrasound diagnostic instrument (Xuzhou Kaixin Electronic Instrument, Jiangsu, China) to accurately measure the back fat thickness of sows at 107 days of gestation, 7 days and 21 days of lactation, and calculate the changes in back fat thickness and body weight of each sow. Record the reproductive performance of sows after farrowing (number of healthy piglets, number of stillbirths).

### 2.4 Sample Collection

Fresh fecal samples were collected directly by rectal massage on days 7 and 21 of lactation, and then immediately stored at −80°C until further analysis. Fasting blood samples were collected from sows through the marginal ear vein on days 7 and 21 of lactation. One piglet from each litter was randomly selected for weaning and fasting overnight on the evening on day 21. The piglets were euthanized by electric shock in the early 22nd day of age. Blood was collected from piglets, organs were weighed, and parts of the liver and thymus were fixed with paraformaldehyde buffer. The liver was immediately snap-frozen in liquid nitrogen and stored at−80 °C for analysis. Plasma was centrifuged at 1,500 × *g* for 10 min at 4 °C and stored at−80 °C for chemical analysis.

### 2.5 Chemical analysis of blood indicators

A glucose oxidase assay kit was used to determine the plasma glucose concentration in sows' fasting blood according to the manufacturer's (Nanjing Jiancheng Bioengineering Institute, Jiangsu, China). Porcine insulin was measured using a porcine ELISA kit (Jiangsu Meimian Industry Co. LTD., Jiangsu, China) was used to determine plasma insulin levels in sows. Insulin resistance and sensitivity were assessed using the homeostatic model: homeostatic model assessment for insulin resistance (HOMA-IR) = fasting insulin (mIU/L) × fasting glucose (mmol/L)/22.5 (coefficient 22.5, a correction factor). The formula for the insulin sensitivity index (ISI) and triglyceride glucose index (TyG) index was as follows: ln (ISI) = ln (1/[fasting glucose (mmol/L) × fasting insulin (mU/L)). TyG= ln (fasting glucose (mg/dL) × fasting triglyceride (mg/dL)/2) (24–26).

The plasma biochemical parameters were measured with a biochemistry auto-analyzer (Hitachi 7,160, Japan), including triglycerides (TG), total cholesterol (T-CHO), total bile acids (TBA), blood urea nitrogen (BUN), glycosylated serum proteins (GSP), uric acid (UA), aspartate transaminase (AST), alanine aminotransferase (ALT), high-density lipoprotein-cholesterol (HDL-C), low density lipoprotein-cholesterol (LDL-C), cholinesterase (CHE), and γ-glutamyl transferase (γ-GT). Levels of free fatty acids (FFA), fibroblast growth factor 21 (FGF21), pyruvate carboxylase (PC), phosphoenolpyruvate carboxykinase (PEPCK), glucose-6-phosphate dehydrogenase (G6PD), and glycogen synthase (GS) were analyzed using enzyme-linked immunosorbent assay (ELISA) kits according to the (Shanghai Enzyme-linked Biotechnology Co., Ltd., Shanghai, China) guidelines provided by the manufacturer.

All experimental diets were formulated to meet the nutrient requirements for sows. Chemical concentrations were calculated using the values for feed ingredients from the National Research Council (78).

### 2.6 Metabolomic profiling analysis

Plasma from sows at day 21 of lactation was used for metabolomics analysis. Chromatographic separations used an ultra-performance liquid chromatography system (SCIEX, Macclesfield, UK.). Metabolites eluted from the column were detected using a high-resolution tandem mass spectrometer TripleTOF56001 (SCIEX) in both positive and negative ion modes. Only the metabolites with a fold change>2or < 0.5 and *P* value < 0.05 were analyzed.

### 2.7 Gut microbiome analysis

Total DNA from colon content samples was extracted using the Qubit dsDNA HS Assay Kit (Invitrogen). The DNA concentration and integrity were measured, and the V3–V4 region of the bacterial 16S rDNA was amplified using PCR. Purification and quantification of the PCR products were performed via AMPure XT beads and Qubit (Invitrogen), respectively. Gut microbiome analysis was performed via high-throughput 16S rDNA sequencing.

### 2.8 Histological examination

Piglet liver and thymus sections were preserved in 4% paraformaldehyde solution for histological analysis. Hematoxylin (Solarbio, China) and eosin (Sangon, China) (H&E) staining was used for histological examination. The resulting tissue blocks were then cut into thin slices of 4 μm thickness, which were subsequently placed on slides and stained. Finally, images were captured using a microscope (OLYMPUS, Japan).

### 2.9 Transcriptomic analysis

Piglet liver tissue was used for transcriptomic analysis. TRIzol reagent (Invitrogen, Carlsbad, CA) was used to isolate and purify the total RNA. The 2 × 150 bp paired-end sequencing was conducted via the Illumina Novaseq 6,000 (LC-Bio Technology Co., Ltd, Hangzhou, China). Only the mRNAs with a fold change >1.5 or < 0.67 and *P* value < 0.05 were chosen to as differentially expressed genes (DEGs) through R package DESeq2.

### 2.10 Gene expression verification

Total RNA in the liver was extracted using TRIzol (Invitrogen) with an A260/A280 ratio between 1.8 and 2.0. cDNA was collected through reverse transcription via the PrimeScript RT reagent kit (Takara). SYBR Green dyes (Takara) were utilized for qPCR. The ABI PRISM 7,500 real-time thermal cycler apparatus system (Applied Biosystems, California, USA) was used for real-time PCR analysis. Quantification of mRNA expression was performed according to the previously described quantitative RT-PCR (qRT-PCR) method (27). Sequences of the selected primers are shown in Supplementary Table S2. A comparative method (2^−Δ*ΔCt*^) was performed to normalize the target gene using β-actin.

### 2.11 Western blotting

Specific operations of Western blotting were carried out as described in previous work (28). Briefly, total protein was extracted from frozen liver tissue in a lysis buffer with protease inhibitors. The proteins were loaded for gel electrophoresis and transferred to polyvinylidene fluoride membranes. Membranes were blocked for 1 h with 5% skim-milk at room temperature in TBS supplemented with 0.1% Tween-20 (TBST). Following this, the membranes were incubated with the following primary antibodies overnight at 4 °C and visualized using chemiluminescence with an enhanced detection system (Biosharp, China). The images were captured with the Advanced Q9 Alliance system (UVITEC, England) and analyzed using the ImageJ system. To standardize the band intensities, β-actin was employed.

### 2.12 Statistical analysis

Each sow and piglet served as a separate experimental unit. All data of the experiment were performed by two-way analysis of variance using SPSS 27.0 (SPSS Inc., Chicago, USA), with “oil level” and “leucine level” as main effects. The statistical model used was as follows: *Y*_*ij*_ = μ+*E*_*i*_+*S*_*j*_+*R*_*ij*_+ε_*ij*_, Where *Y*_*ij*_ is the observation of dependent variables; μ is the overall mean; *E*_*i*_ is the “oil level” effect; *S*_*j*_ is the “leucine level” effect, *R*_*ij*_ represents the interactive effect between the two factors, and ε_*ij*_ is the residual error for the observation. Meanwhile, differences among the 4 treatments were evaluated using two-way ANOVA and Duncan's multiple comparisons when a significant interaction was observed. Results are expressed as mean and pooled standard error of the mean (SEM) and considered as significant at *P* < 0.05.

## 3 Results

### 3.1 Productive performance, reproductive performance and feed intake of sows in late gestation and lactation

As shown in Supplementary Table S3, HSOL group exhibited significantly reduced sow body weights at both L7d (*P* = 0.006) and L21d (*P* = 0.001). From G107d to L7d, HSO group showed significantly decreased weight loss (*P* < 0.001), while HSOL group displayed an opposite trend. Significant soybean oil × leucine interactions were observed at L7d (*P* = 0.006), L21d (*P* = 0.001) and during G107d-L7d (*P* < 0.001). Soybean oil supplementation significantly increased backfat thickness at L21d (*P* = 0.018). Furthermore, CL, HSO and HSOL groups exhibited increased backfat during L7d-L21d phase (*P* < 0.001), with significant oil × leucine interaction (*P* = 0.002) at this stage.

### 3.2 Plasma biochemical parameters in sows at 7 and 21 days of lactation

Table 1 details changes in plasma biochemical indicators of sows over time. At L7d, plasma TG content significantly increased in CL and HSOL groups compared to group C (*P* = 0.004). Plasma insulin levels significantly decreased in the HSO group (*P* = 0.002). Plasma FGF21 concentration was significantly reduced in groups supplemented with fat or leucine alone (HSO and CL; *P* = 0.034 and *P* = 0.032, respectively). At L21d, plasma glucose and PC levels significantly increased in group CL (*P* = 0.036). Glucose levels were affected by both fat and leucine, with a highly significant interaction (*P* < 0.001). The HSOL group exhibited a significant increase in plasma FFA (*P* = 0.037) and a significant decrease in plasma PC (*P* = 0.020). Plasma GS levels increased significantly in all treatment groups (CL, HSO, HSOL; *P* = 0.024, *P* = 0.002, *P* = 0.039). No significant differences (*P* > 0.05) were found for TG, TC, LDL, HDL, ALT, AST, GSP, INS, TBA, CHE, γ-GT, BUN, UA, FGF21, PEPCK, or G6PD levels/activity.

**Table 1:** Effects on plasma biochemical parameters in sows at 7 and 21 days of lactation.

Item	Treatment				SEM	*P*–value		
	C	CL	HSO	HSOL		O	L	O × L
L7 d								
TG (mmol/L)	0.25^ab^	0.30^a^	0.22^b^	0.29^a^	0.010	0.369	0.004	0.624
TC (mmol/L)	3.39	3.84	3.71	3.65	0.151	0.835	0.520	0.394
LDL (mmol/L)	1.41	0.99	1.90	1.03	0.208	0.533	0.133	0.585
HDL (mmol/L)	1.61	1.41	1.83	1.56	0.130	0.471	0.380	0.890
ALT (U/L)	11.57	8.34	7.10	13.18	1.448	0.951	0.627	0.121
AST (U/L)	20.02	23.77	6.99	7.46	2.706	0.012	0.700	0.764
GLU (mmol/L)	2.33	3.11	2.78	2.99	0.153	0.581	0.118	0.360
GSP (mmol/L)	1.38	1.44	1.44	1.40	0.051	0.865	0.921	0.623
INS (mIU/L)	29.78^a^	28.87^ab^	25.15^c^	25.90^bc^	0.533	0.002	0.938	0.446
FFA (μmol/mL)	0.95	1.09	0.62	0.69	0.094	0.066	0.582	0.850
TBA (μmol/L)	9.48	12.29	7.96	7.20	0.854	0.063	0.553	0.305
CHE (U/L)	27.87	17.27	24.00	20.80	2.611	0.975	0.198	0.485
γ-GT (U/L)	46.75	56.02	51.14	44.36	3.179	0.572	0.847	0.218
BUN (mmol/L)	1.44	1.67	1.65	1.30	0.109	0.714	0.763	0.193
UA (μmol/L)	67.21	49.89	41.56	43.24	5.616	0.161	0.492	0.405
FGF21 (ng/L)	209.30^a^	172.63^b^	173.18^b^	181.77^ab^	5.431	0.235	0.217	0.056
L21 d								
TG (mmol/L)	0.24	0.25	0.22	0.20	0.015	0.200	0.966	0.556
TC (mmol/L)	4.15	4.89	5.01	4.60	0.182	0.435	0.648	0.125
LDL (mmol/L)	2.19	1.76	3.40	1.69	0.293	0.333	0.079	0.284
HDL (mmol/L)	2.10	2.16	2.01	2.29	0.131	0.944	0.521	0.675
ALT (U/L)	18.62	16.14	13.56	6.73	2.534	0.165	0.367	0.671
AST (U/L)	7.61	8.59	10.36	9.05	1.277	0.534	0.948	0.658
GLU (mmol/L)	0.89^b^	2.64^a^	1.41^b^	0.96^b^	0.106	0.011	0.005	< 0.001
GSP (mmol/L)	1.24	1.31	1.39	1.22	0.028	0.579	0.375	0.046
INS (mIU/L)	26.67	27.75	26.64	30.48	0.750	0.384	0.120	0.371
FFA (μmol/mL)	0.85^b^	0.56^b^	1.10^b^	1.88^a^	0.119	0.004	0.306	0.037
TBA (μmol/L)	20.04	20.68	11.18	10.75	1.805	0.015	0.979	0.883
CHE (U/L)	22.87	30.18	19.33	26.93	3.057	0.585	0.235	0.981
γ–GT (U/L)	49.94	50.49	50.71	37.65	3.497	0.396	0.379	0.340
BUN (mmol/L)	2.99	3.24	2.23	2.92	0.226	0.241	0.307	0.638
UA (μmol/L)	41.88	47.13	32.63	33.17	2.926	0.057	0.625	0.691
FGF21 (ng/L)	178.87	168.84	163.24	175.60	4.575	0.632	0.900	0.233
PC (U/L)	226.02^b^	249.57^a^	205.64^bc^	191.79^c^	3.708	< 0.001	0.521	0.020
PEPCK (IU/L)	165.06	180.85	167.12	182.63	4.588	0.836	0.104	0.988
G6PD (U/L)	189.17	176.77	189.08	177.71	3.132	0.946	0.072	0.936
GS (U/L)	754.20^a^	570.45^b^	604.77^b^	563.11^b^	15.843	0.024	0.002	0.039

Data were presented as means with SEM (n = 8).

^a−c^Different shoulder markers represent significant differences (*P* < 0.05).

C, control diet group; CL, control + 1% leucine diet group; HSO, high soybean oil group; HSOL, high soybean oil+ 1% leucine diet group; O, supplement soybean oil; L, supplement leucine; O × L, interactions between soybean oil supplementation and leucine; SEM, standard error of the means; L7 d, lactation day 7; L21 d, lactation day 21; TG, triglyceride; TC, total cholesterol; LDL, low–density lipoprotein; HDL, high–density lipoprotein; ALT, alanine aminotransferase; AST, aspartate aminotransferase; GLU, glucose; GSP, glycated serum protein; INS, insulin; FFA, free fatty acid; TBA, total bile acid; CHE, cholinesterase; γ-GT, gamma–glutamyl transferase; BUN, blood urea nitrogen; UA, uric acid; FGF21, fibroblast growth factor 21; PC, pyruvate carboxylase; PEPCK, phosphoenolpyruvate carboxykinase; G6PD, glucose−6–phosphate dehydrogenase; GS, glycogen synthase.

### 3.3 Assessment of blood glucose homeostasis and insulin sensitivity in sows at 107 days of gestation, 7 days of lactation, and 21 days of lactation

Glucose tolerance tests were carried out on sows at 107 days of gestation and 7 days of lactation. The results showed that at each time point, there were no significant differences in the area under the blood glucose curve (*P* > 0.05) (Supplementary Figure S1A and Supplementary Figure S1B). The HOMA-IR, ISI and TyG index of sows on day 7 and day 21 of lactation were calculated (Table 2). On L7d, dietary soybean oil significantly impacted all indices, sows receiving HSO had significantly lower HOMA-IR and TyG indices compared to the C group (*P* = 0.015 and *P* = 0.028), while their ISI index was significantly higher (*P* = 0.021). Sows receiving leucine alone (CL) or the combination (HSOL) had intermediate values that did not differ significantly from each other or consistently from the C and HSO as indicated by the letter superscripts. On L21d, both dietary soybean oil (O) and leucine (L), along with their interaction (O × L), showed significant effects on various indices. The CL group exhibited a significantly elevated HOMA-IR compared to all other groups (*P* = 0.005). The HSOL group showed a significantly lower TyG index compared to groups C and CL, and the lowest HOMA-IR value (*P* = 0.036 and *P* = 0.006). Sows receiving soybean oil (groups HSO and HSOL) generally had significantly lower TyG and HOMA-IR indices compared to those receiving no soybean oil (groups C and CL) (*P* < 0.001 and *P* = 0.036), while also showing higher ISI values (HSOL having the highest) (*P* = 0.004).

**Table 2:** Insulin resistance index, insulin sensitivity index, and triglyceride–glucose index of sows on days 7 and 21 of lactation.

Item	Treatment				SEM	*P*–value		
	C	CL	HSO	HSOL		O	L	O × L
L7d								
HOMA–IR	3.63^a^	3.02^ab^	2.59^b^	2.74^b^	0.116	0.015	0.344	0.131
ln (ISI)	−4.39^b^	−4.22^ab^	−4.06^a^	−4.10^a^	0.042	0.021	0.423	0.233
TyG	6.54^a^	6.39^ab^	5.96^b^	6.26^ab^	0.071	0.028	0.608	0.147
L21d								
HOMA-IR	1.68^b^	3.02^a^	1.14^bc^	0.70^c^	0.130	< 0.001	0.108	0.006
ln (ISI)	−3.59^b^	−4.17^b^	−3.21^ab^	−2.40^a^	0.153	0.004	0.724	0.042
TyG	5.51^a^	5.72^a^	4.99^ab^	3.37^b^	0.303	0.036	0.268	0.158

Data were presented as means with SEM (n = 8).

^a−c^Different shoulder markers represent significant differences (*P* < 0.05).

C, control diet group; CL, control + 1% leucine diet group; HSO, high soybean oil group; HSOL, high soybean oil+ 1% leucine diet group; O, supplement soybean oil; L, supplement leucine; O × L, interactions between soybean oil supplementation and leucine; SEM, standard error of the means; L7 d, lactation day 7; L21 d, lactation day 21; HOMA–IR, homeostatic model assessment for insulin resistance; ISI, insulin sensitivity index; TyG, triglyceride and glucose index.

### 3.4 Plasma metabolomics analysis

**Figure 1:**
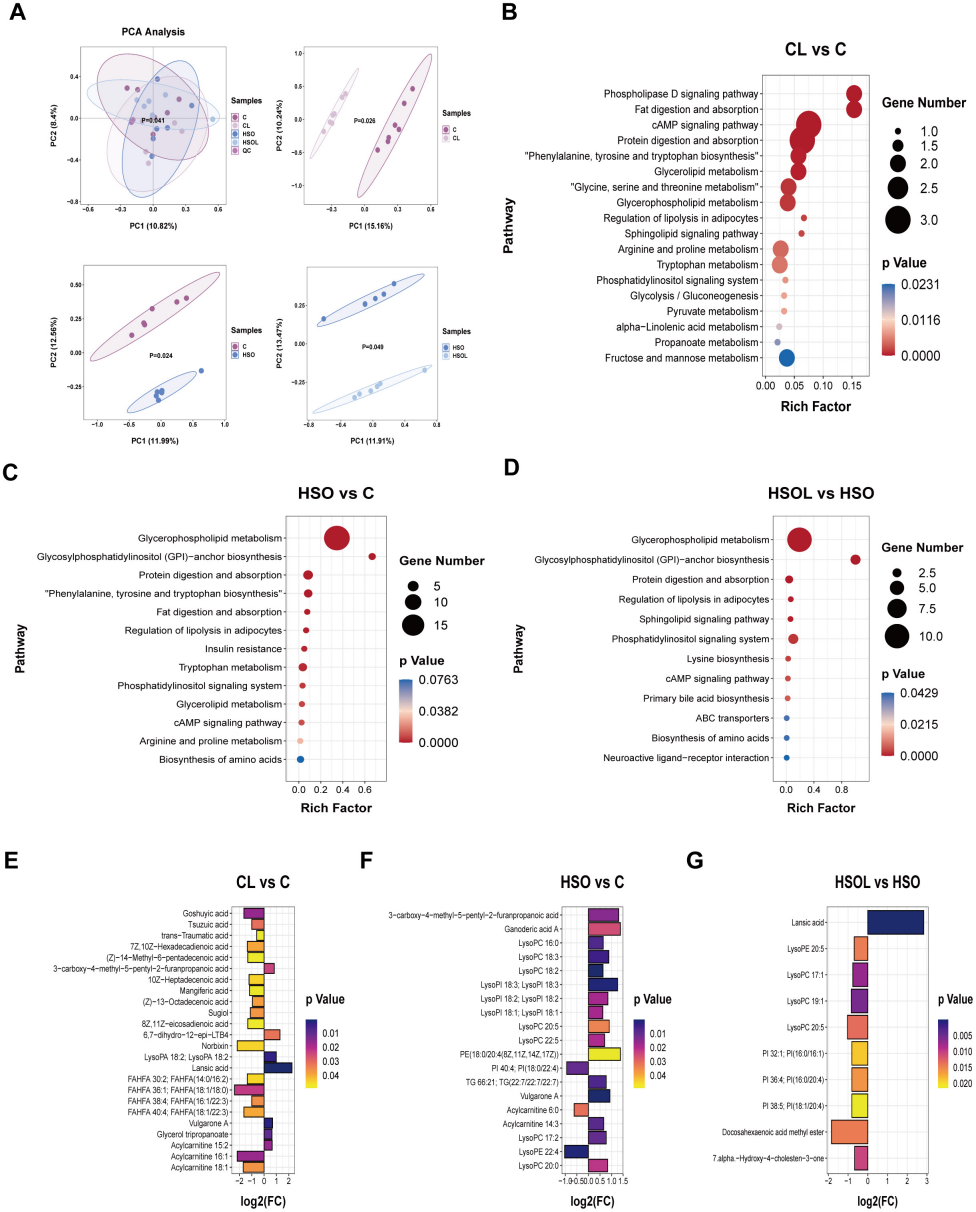
Serum metabolomics analysis of sows on day 21 of lactation. **(A)** PCA analysis of all metabolites and PLS-DA analysis of comparative groups (*n* = 6). **(B–D)** KEGG functional enrichment analysis of differential metabolites in serum of sows on day 21 of lactation (n = 6). **(E–G)** Changes in secondary metabolites in serum of sows at 21 days of lactation that are significantly different between comparison groups (*n* = 6). C, Control diet group; CL, Control + 1% leucine diet group; HSO, High soybean oil group; HSOL, High soybean oil+ 1% leucine diet group; PCA, Principal component analysis; PLS–DA, Partial least squares discriminant analysis.

Plasma metabolomics analysis of lactating sows on day 21 identified significant intergroup metabolic variation (Figure 1). Principal Coordinates Analysis (PCoA) demonstrated significant separation of plasma metabolite profiles in all treatment groups relative to the control following supplementation with soybean oil and leucine (*P* = 0.026, *P* = 0.024, *P* = 0.049; Figure 1A). KEGG pathway enrichment analysis revealed distinct metabolic alterations compared to group C: CL Group, pathways showing significant enrichment were predominantly related to lipid metabolism (including phospholipase D signaling, alpha-linolenic acid metabolism, glycerophospholipid metabolism, glycerolipid metabolism, fat digestion and absorption, regulation of lipolysis in adipocytes), amino acid metabolism (phenylalanine/tyrosine/tryptophan biosynthesis, glycine/serine/threonine metabolism, arginine/proline metabolism, tryptophan metabolism), and carbohydrate metabolism (fructose and mannose metabolism, glycolysis/gluconeogenesis) (Figure 1B). HSO Group enrichment was primarily observed in pathways involved in lipid metabolism (glycerophospholipid metabolism, glycerolipid metabolism, fat digestion and absorption, regulation of lipolysis in adipocytes, phosphatidylinositol signaling system, glycosylphosphatidylinositol (GPI)-anchor biosynthesis) and the insulin resistance pathway (Figure 1C). HSOL Group enrichment covered multiple lipid metabolism pathways and uniquely featured primary bile acid biosynthesis (Figure 1D). Analysis of secondary metabolite composition highlighted specific alterations: CL vs. C: Differential metabolites were principally characterized by increased levels of fatty acid esters of hydroxy fatty acids (FAHFAs) and acylcarnitines (Figure 1E). HSO vs. C: Metabolites exhibiting significant alterations belonged to the lysophosphatidylcholine (LysoPC), acylcarnitine, and lysophosphatidylinositol (LysoPI) families (Figure 1F). HSOL vs. HSO: Differential metabolites were mainly concentrated in LysoPC and phosphatidylinositol (PI) classes (Figure 1G). Collectively, these data indicate that dietary intervention with soybean oil and leucine significantly alters porcine lipid metabolism pathways and associated secondary metabolite profiles during lactation.

### 3.5 Dietary supplementation with soybean oil and leucine altered the fecal microbiota structure in lactating sows

**Figure 2:**
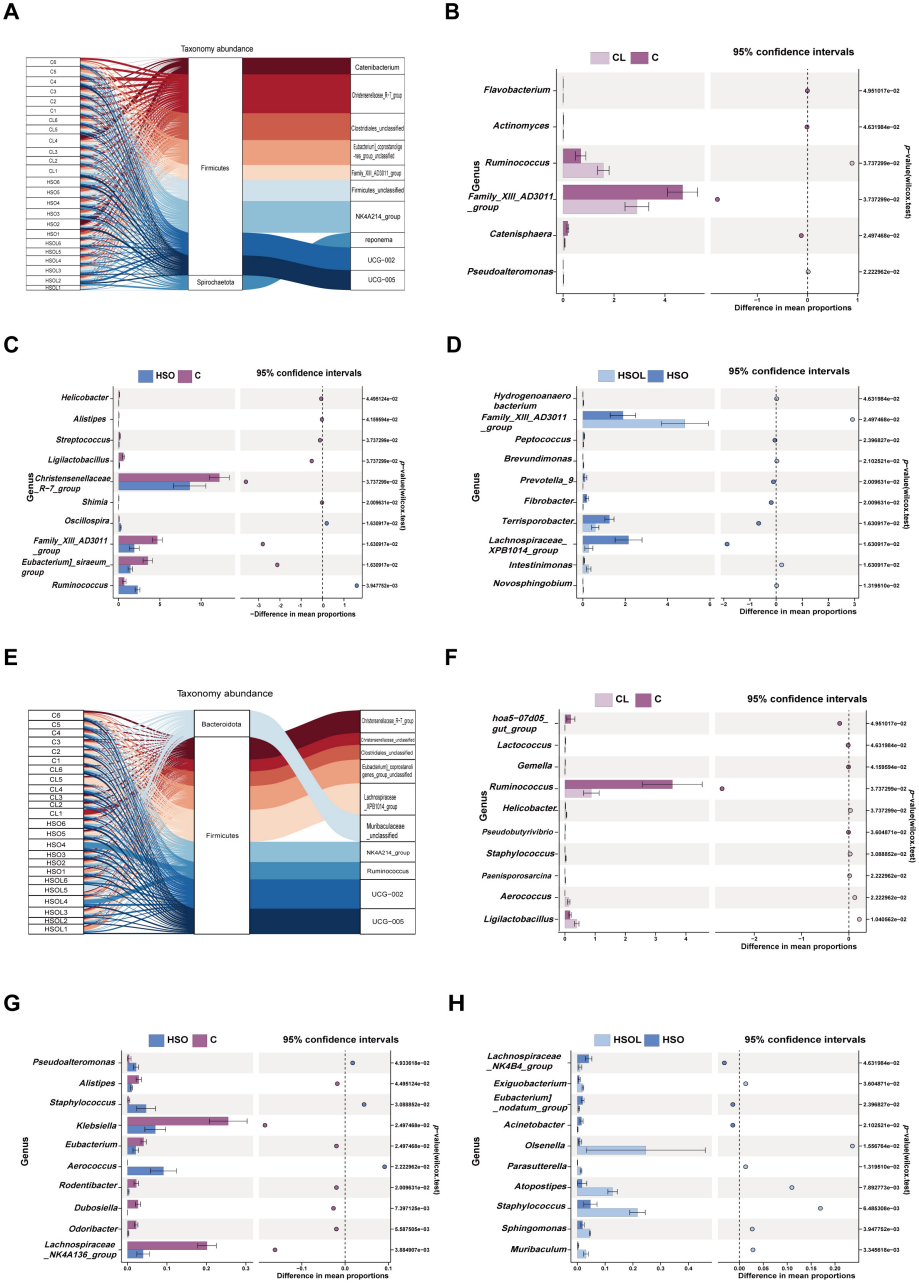
Fecal microbiota composition of sows at days 7 and 21 of lactation. **(A)** Sankey plot of the top 10 different intestinal bacteria and their phylum levels at the genus level at 7 days of lactation(*n* = 6); **(B–D)** Stamp analysis plots of the top 10 bacterial genera with significant differences in each comparison group at 7 days of lactation(*n* = 6); **(E)** Sankey plot of the top 10 different intestinal bacteria and their phylum levels with significant differences at the genus level at 21 days of lactation(*n* = 6); **(F–H)** Stamp analysis plots of the top 10 bacterial genera with significant differences in each comparison group at 21 days of lactation (*n* = 6). C, Control diet group; CL, Control + 1% leucine diet group; HSO, High soybean oil group; HSOL, High soybean oil+ 1% leucine diet group.

At L7d, microbial composition was significantly influenced by genera affiliated with *Firmicutes* and *Spirochaetota* (*P* < 0.05; Figure 2A). Analysis of the top 10 differentially abundant genera revealed distinct treatment effects: The CL group exhibited depletion of *Family_XIII_AD3011_group, Catenisphaera, Actinomyces*, and *Flavobacterium* with concurrent enrichment of *Ruminococcus* and *Pseudoalteromonas* (Figure 2B). In contrast, the HSO group showed depletion of *Christensenellaceae_R-7_group, Family_XIII_AD3011_group, Eubacterium_siraeum_group, Ligilactobacillus, Streptococcus, Helicobacter*, and *Alistipes* alongside enrichment of *Ruminococcus* and *Oscillospira* (Figure 2C). Meanwhile, the HSOL group displayed significant depletion of *Lachnospiraceae_XPB1014_group, Terrisporobacter, Fibrobacter, Prevotella_9* and *Peptococcus*, accompanied by enrichment of *Family_XIII_AD3011_group, Intestinimonas, Brevundimonas, Hydrogenoanaerobacterium* and *Novosphingobium* (*P* < 0.001; Figure 2D). Notably, leucine supplementation (CL) specifically altered *Family_XIII_AD3011_group* abundance, whereas soybean oil supplementation (HSO) significantly modulated *Ruminococcus*.

By L21d, predominant differentially abundant genera belonged to Firmicutes and Bacteroidota (*P* < 0.01; Figure 2E). Compared to C, the CL group demonstrated reduced relative abundance of *Ruminococcus, hoa5-07d05_gut_group, Lactococcus, Pseudobutyrivibrio* and *Gemella*, but elevated levels of *Ligilactobacillus, Aerococcus, Helicobacter, Staphylococcus*, and *Paenisporosarcina* (Figure 2F). The HSOL group (vs. C) exhibited depletion of *Klebsiella, Lachnospiraceae_NK4A136_group, Eubacterium, Alistipes, Dubosiella, Rodentibacter* and *Odoribacter*, coupled with enrichment of *Aerococcus, Staphylococcus*, and *Pseudoalteromonas* (Figure 2G). Furthermore, relative to the HSO group, HSOL induced depletion of *Lachnospiraceae_NK4B4_group, Eubacterium_nodatum_group* and *Acinetobacter* with concomitant enrichment of *Staphylococcus, Olsenella, Atopostipes, Sphingomonas, Muribaculum, Exiguobacterium* and *Parasutterella* (Figure 2H). Critically, individual supplementation with either soybean oil (CL group) or leucine (HSO group) significantly enriched *Aerococcus* and *Staphylococcus* abundance compared to control (*P* < 0.001).

### 3.6 Piglet growth performance and organ development

Building upon the maternally-derived benefits of soybean oil and leucine supplementation, growth performance of piglets (Table 3) demonstrated: At 14d, PHSOL litters significantly exceeded PC (*P* = 0.021). PCL with elevated individual weight at day 14 (*P* = 0.015). At 21d, progressive increase across treatments, soybean oil and leucine independently and additively enhanced 21-day weights of piglets (*P* < 0.001 and *P* < 0.001). Organ assessment (Table 4) showed: PCL exhibited reduced pancreas-to-body weight ratios (*P* = 0.022). All supplemented groups (PCL/PHSO/PHSOL) increased thymus indices relative to PC (*P* = 0.013).

**Table 3:** Effects of soybean oil and leucine in sow diets on growth performance of piglets.

Item	Treatment				SEM	*P*–value		
	PC	PCL	PHSO	PHSOL		O	L	O × L
Litter weight of piglets, kg								
birth weight	20.79	17.22	20.47	18.61	0.755	0.730	0.087	0.577
14 d	38.54^b^	43.69^ab^	44.20^ab^	47.23^a^	1.224	0.075	0.110	0.669
21 d	57.62	60.04	64.79	63.28	1.268	0.054	0.858	0.447
Weight of piglets, kg								
birth weight	1.49	1.55	1.48	1.39	0.048	0.410	0.904	0.446
14 d	3.34^b^	4.05^a^	3.57^ab^	3.95^ab^	0.103	0.749	0.015	0.427
21 d	4.85^a^	5.58^b^	5.90^c^	6.38^d^	0.039	< 0.001	< 0.001	0.120

Data were presented as means with SEM (n = 6).

^a−c^Different shoulder markers represent significant differences (*P* < 0.05).

PC, piglets born and fed by sows in group C; PCL, piglets born and fed by sows in group CL; PHSO, piglets born and fed by sows in group HSO; PHSOL, piglets born and fed by sows in group HSOL; O, supplement soybean oil; L, supplement leucine; O × L, interactions between soybean oil supplementation and leucine; SEM, standard error of the means; 14d, the 14^th^ day after the birth of piglets; 21d, the 21^th^ day after the birth of piglets.

**Table 4:** Relative organ weights of various organs and Insulin resistance index, insulin sensitivity index, and triglyceride glucose index of piglets.

Item	Treatment				SEM	*P*–value		
	PC	PCL	PHSO	PHSOL		O	L	O × L
Relative organ weight, g/kg								
heart	6.56	6.06	6.26	6.97	0.122	0.234	0.661	0.022
liver	25.12	24.93	23.91	24.35	0.561	0.434	0.907	0.781
spleen	1.91	1.65	2.19	2.12	0.077	0.023	0.303	0.566
lung	14.60	14.77	15.54	15.47	0.343	0.243	0.943	0.865
kidney	6.11	5.72	6.69	5.75	0.168	0.375	0.062	0.415
pancreas	1.18^a^	0.66^b^	1.04^ab^	0.76^ab^	0.074	0.881	0.015	0.412
thymus	0.84^b^	1.55^a^	1.52^a^	1.98^a^	0.107	0.018	0.013	0.555
lnguinal lymph node	0.65	0.44	0.63	0.49	0.034	0.819	0.016	0.678
Index								
HOMA–IR	6.55^a^	5.12^ab^	5.65^ab^	3.71^b^	0.345	0.121	0.031	0.720
ln (ISI)	−4.99	−4.73	−4.84	−4.05	0.159	0.219	0.124	0.425
TyG	8.01	7.11	7.63	7.04	0.195	0.569	0.080	0.696

Data were presented as means with SEM (n = 6).

^a − c^Different shoulder markers represent significant differences (*P* < 0.05). PC, piglets born and fed by sows in group C; PCL, piglets born and fed by sows in group CL; PHSO, piglets born and fed by sows in group HSO; PHSOL, piglets born and fed by sows in group HSOL; O, supplement soybean oil; L, supplement leucine; O × L, interactions between soybean oil supplementation and leucine; SEM, standard error of the means; HOMA–IR, homeostatic model assessment for insulin resistance; ISI, insulin sensitivity index; TyG, triglyceride and glucose index.

### 3.7 Plasma biochemical parameters and assessment of insulin homeostasis in piglets

Maternal supplementation with soybean oil and leucine significantly modulated offspring plasma biochemistry and insulin homeostasis. The oil-leucine interaction elevated triglycerides in PHSOL piglets (*P* = 0.033). PHSOL piglets exhibited higher plasma free fatty acids than PHSO counterparts (*P* = 0.048). Maternal CL treatment reduced offspring FGF21 concentrations (*P* = 0.039) (Supplementary Table S4). HSOL treatment lowered the HOMA-IR index in weaned piglets, mainly due to the influence of leucine (*P* = 0.031) (Table 4).

### 3.8 Morphological observation of liver and thymus tissues in piglets

Maternal supplementation differentially impacted offspring liver (Supplementary Figure S2A) and thymus (Supplementary Figure S2B) histomorphology. Hematoxylin-eosin staining of liver and thymus was performed. In the PCL group, hepatocytes were mildly enlarged in size, cellular swelling, and the structure of the lobules became unclear. In the PHSO group, macro vesicular steatosis was observed (red arrow), and single lipid droplet vacuoles or a few rounded lipid droplet vacuoles appeared in the cytoplasm of hepatocytes. Histological analysis of the thymus showed that the density of cortical thymocytes was reduced in the PC group, with an enlarged gap (black arrow) around the cortical and medullary union, the thymus organ index was increased in all the experimental groups.

### 3.9 Hepatic transcriptome profiling identified maternally transferred metabolic effects in piglets

**Figure 3:**
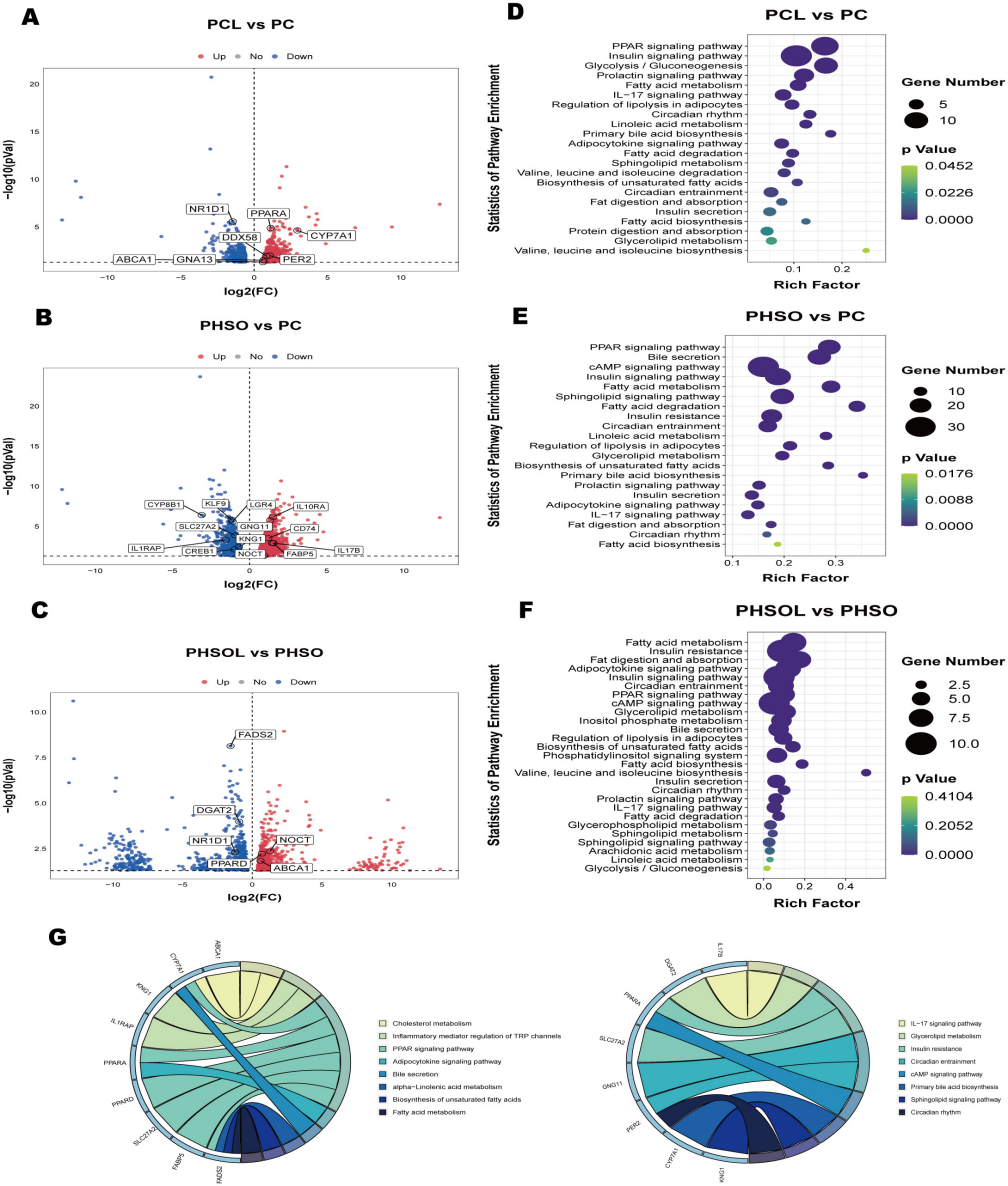
Expression of genes related to lipid metabolism, inflammation and immunity, rhythm in piglets. **(A–C)** Volcano plots of DEGs in the liver of each comparison group of piglets; **(D–F)** KEGG enrichment analysis of DEGs in the liver of each comparison group of piglets; **(G)** String plot of the relationship between differentially expressed genes related to lipid metabolism, immunity, and rhythm in piglets and KEGG functions (*n* = 6). PC, Piglets born and fed by sows in group C; PCL, Piglets born and fed by sows in group CL; PHSO, Piglets born and fed by sows in group HSO; PHSOL, Piglets born and fed by sows in group HSOL.

RNA-seq analysis revealed differential expression of lipid metabolism-related genes across all comparison groups. KEGG enrichment consistently highlighted PPAR signaling, prolactin signaling, fatty acid metabolism, IL-17 signaling, adipocyte lipolysis regulation, circadian rhythm, linoleic acid metabolism, fat digestion/absorption, insulin secretion, and glycerolipid metabolism as core enriched pathways (Figures 3D–F). Focused screening identified key DEGs, Lipid metabolism: *ABCA1, FADS2, FABP5, SLC27A2, PPARA, PPARD, DGAT2, CYP7A1, CYP8B1, CREB1*, Inflammation/immunity: *IL1RAP, KNG1, IL17B, IL10RA, CD74, DDX58, GNA13* and Circadian regulation: *GNG11, PER2, LGR4, NOCT, KLF9, NR1D1* (Figures 3A–C). These DEGs represent potential molecular mediators conveying maternal supplementation benefits (Figure 3G).

### 3.10 Validation of key DEGs in piglet liver tissue

**Figure 4:**
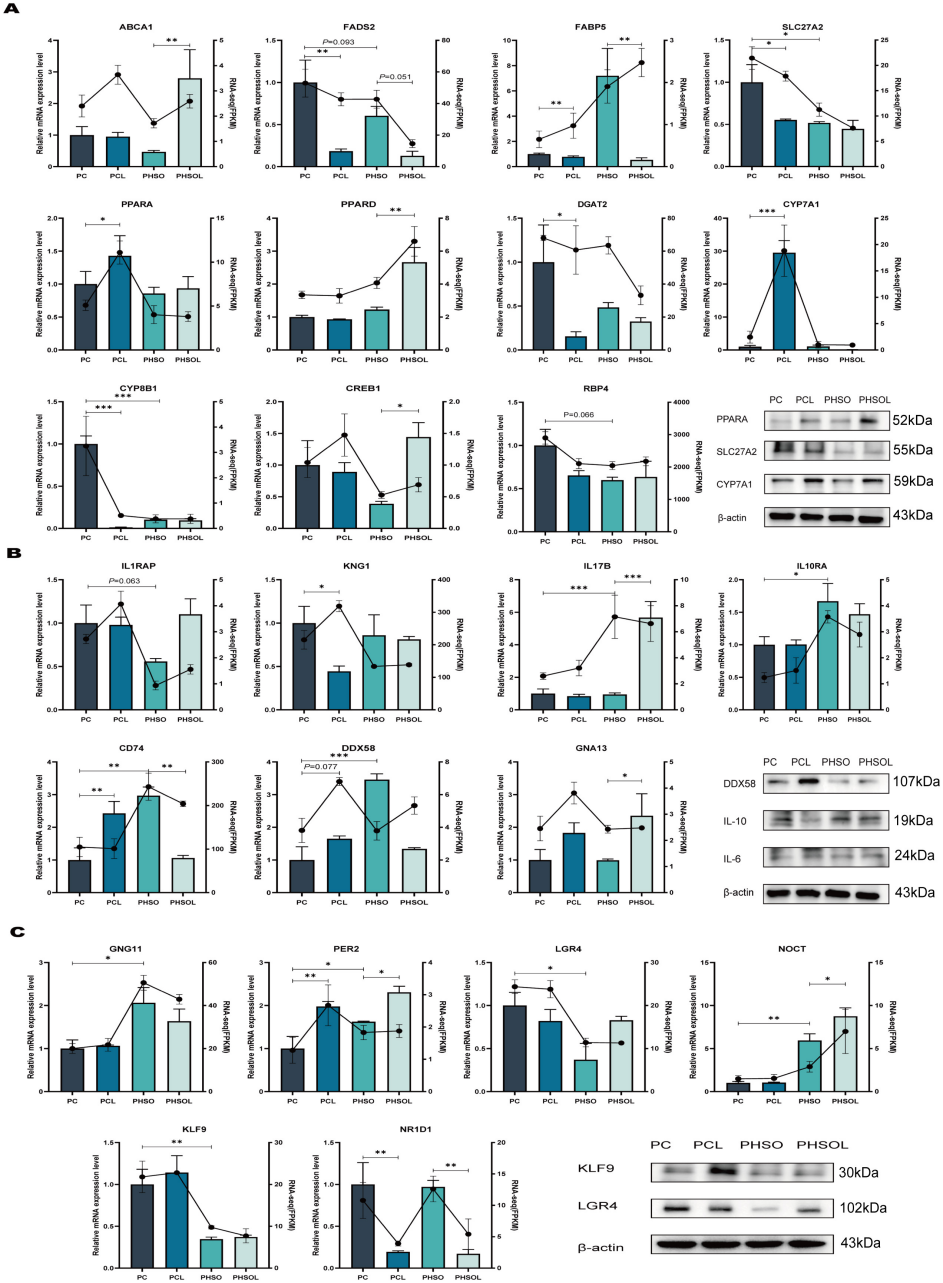
Validation of the effects of lipid metabolism, inflammation and immunity, circadian rhythms on mRNA and protein levels in piglets. **(A)** qRT–PCR validation of RNA sequencing related to lipid metabolism in piglet liver (*n* = 6) and protein expression (*n* = 3). **(B)** qRT–PCR validation of RNA sequencing related to inflammation and immunity in piglet liver (*n* = 6) and protein expression (*n* = 3). **(C)** qRT–PCR validation of RNA sequencing related to circadian rhythm in piglet liver (*n* = 6) and protein expression (*n* = 3). PC, Piglets born and fed by sows in group C; PCL, Piglets born and fed by sows in group CL; PHSO, Piglets born and fed by sows in group HSO; PHSOL, Piglets born and fed by sows in group HSOL. The bar graph and line graph represent the results of qRT–PCR and RNA sequencing. ^*^:*P* < 0.05, ^**^:*P* < 0.01, ^***^:*P* < 0.001.

For lipid metabolism-related genes (Figure 4A), PCL treatment decreased *FADS2, SLC27A2, DGAT2*, and *CYP8B1* expression (*P* ≤ 0.026) while elevating *PPARA* and *CYP7A1* (*P* < 0.001). PHSO treatment reduced *SLC27A2* and *CYP8B1* expression (*P* ≤ 0.019) with a trend toward decreased *RBP4* (*P* = 0.066). PHSOL treatment exhibited elevated *PPARD* and *CREB1* (*P* ≤ 0.013) but reduced *FADS2* trend (*P* = 0.051) vs. PHSO.

Concerning inflammation/immunity genes (Figure 4B), PCL group showed increased *CD74* (*P* = 0.009) and trending *DDX58* elevation (*P* = 0.077). PHSO group upregulated *IL10RA* and *CD74* (*P* ≤ 0.025) with *IL1RAP* downregulation trend (*P* = 0.062). PHSOL reduced *CD74* (*P* = 0.029) but elevated *GNA13* (*P* = 0.044) compared to PHSO.

For circadian rhythm genes (Figure 4C), PCL increased *PER2* (*P* = 0.003) while decreasing *NR1D1* (*P* = 0.003). PHSO elevated *GNG11, PER2*, and *NOCT* (*P* ≤ 0.030) but suppressed *LGR4* and *KLF9* (*P* ≤ 0.013). PHSOL decreased *PER2, NOCT* (*P* ≤ 0.021), and *NR1D1* (*P* = 0.004) vs. PHSO. Overall, the qRT-PCR and WB results were basically consistent with the trend of transcriptome in general.

### 3.11 Associations between key physiological indicators in sows at L21d and DEGs in piglets

**Figure 5:**
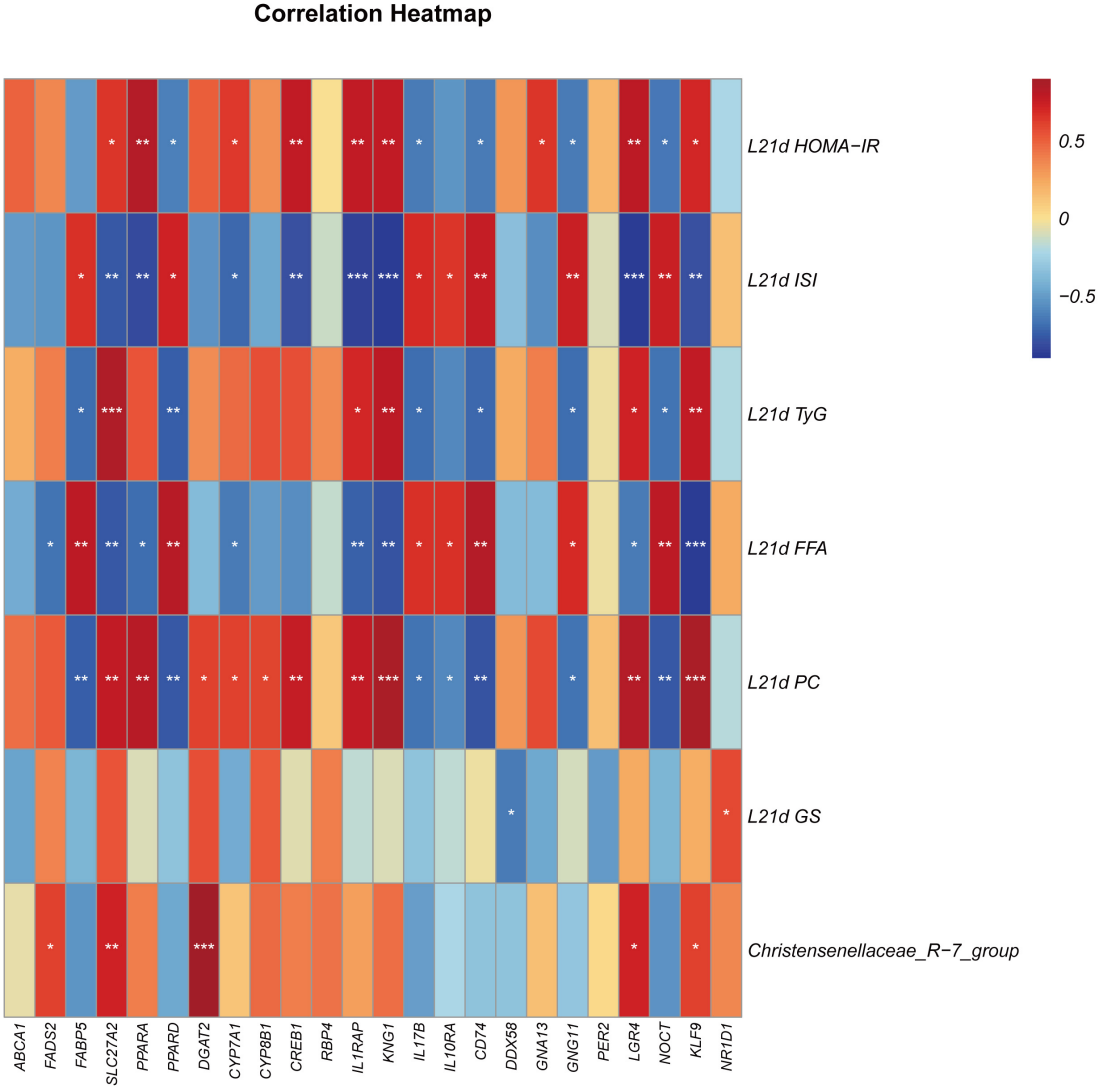
The correlation between physiological indexes of sows on 21 days of lactation and differentially expressed genes of piglets. Crimson red, Strong positive correlation; Dark blue, Strong negative correlation; Light color/no ^*^: weak or insignificant correlation. ^*^:*P* < 0.05, ^**^:*P* < 0.01, ^***^:*P* < 0.001.

As Figure 5 showed, maternal HOMA-IR showed positive correlations with piglet lipid metabolism genes (*PPARA, CREB1*) but negative correlation with the circadian regulator *GNG11* and *NOCT*. Conversely, ISI correlated positively with lipid-related genes *PPARD*, suggesting maternal insulin status modulates offspring lipid pathways. Elevated maternal FFA exhibited strong positive correlations with inflammatory genes (*IL10RA, IL17B, CD74*), indicating potential transmission of metabolic stress to pro-inflammatory responses in piglets. PC levels positively correlated with circadian genes *KLF9* and *LGR4*. Notably, the abundance of *Christensenellaceae_R-7_group* positively associated with both lipid transporter *DGAT2* and circadian genes *LGR4*, implying microbial modulation of lipid- circadian crosstalk. Functionally, lipid metabolism genes predominantly correlated positively with maternal HOMA-IR, while inflammatory genes and circadian regulators showed consistent negative correlations with insulin sensitivity indices (HOMA-IR, TyG), highlighting divergent regulatory patterns across functional gene clusters.

## 4 Discussion

Late gestation and lactation are critical periods, during which they face the highest risk of adverse outcomes during the production period (29). The health status of the maternal is closely related to the growth and health of the offspring (30). Allocation of metabolic energy between milk synthesis and adipose deposition is critically regulated by nutrient signaling. Leucine activates the mTOR signaling pathway to enhance energy expenditure (31). This mechanism is particularly operative in mammary epithelial cells to promotes milk protein and lipid synthesis (32). Simultaneously, leucine modulates lipid metabolism and energy homeostasis, thereby influencing body fat distribution and storage (33). Conversely, soybean oil, prioritizes partitioning toward milk fat synthesis over adipocyte deposition. This preferential allocation likely stems from its constituent fatty acids, which modulate insulin sensitivity and metabolic hormone profiles to redirect energy utilization (34, 35). In the present study, HSOL-treated sows exhibited significantly reduced body mass at L21d compared to C, potentially reflecting leucine-enhanced placental nutrient transfer during late gestation/early lactation (G107d-L7d) (36). This accelerated fetal nutrient provision aligns with established placental fatty acid selectivity (37), contributing to initial maternal mass differences that normalized with prolonged supplementation. While neonatal weights remained comparable across groups, treatment-induced advantages manifested from day 14 onward, yielding significantly heavier weaned piglets—a critical determinant of post-weaning performance (38). Concomitant alterations in milk composition likely augmented this transgenerational effect. Furthermore, dietary fat sustained optimal maternal backfat reserves, a known prerequisite for reproductive efficiency (39), while supporting postpartum recovery. It is generally accepted that swine adjust their voluntary feed intake based on dietary energy density to maintain a constant energy intake. However, in the current study, the increased dietary energy density via soybean oil supplementation did not significantly depress ADFI. This suggests that the substantial energy deficit and high metabolic drive associated with lactation in modern sows may override the chemostatic feedback mechanisms that typically downregulate intake. Consequently, since the dietary CP content was similar across treatments (≈ 16%), the total daily protein intake was maintained in the high-oil groups, preventing protein supply from becoming a limiting factor for performance.

Insulin sensitivity in sows has received increasing attention from researchers (40, 41). TG, INS, and GLU indicate that both fats and leucine influence insulin signaling pathways (42), as quantified through established indices (HOMA-IR, ISI, TyG) where reduced HOMA-IR/TyG and elevated ISI reflect improved sensitivity (43–45). Experiments have shown that dietary fat supplementation improves insulin sensitivity in sows and piglets, which were not seen with leucine alone but trending positively when fats are supplemented together, reflecting a reciprocal effect. Our findings demonstrate soybean oil supplementation enhances insulin sensitivity in sows and progeny—an effect not replicated by leucine alone but potentially augmented through soybean oil-leucine crosstalk. Glucose fluctuations appear mediated by PC and GS activity (46, 47), suggesting coordinated metabolic reprogramming. Although FGF21 correlates with insulin sensitivity across species, our results contrast with rodent models highlighting species-specific mechanisms (48). Importantly, the interplay between BUN and FFA serves as a metabolic nexus for energy partitioning. Their crosstalk may govern energy distribution between protein anabolism and lipid storage (49, 50). Targeted modulation of these metabolic indicators could optimize energy utilization though their intricate interactions warrant further investigation.

Reduced acylcarnitine levels reflect enhanced mitochondrial β-oxidation, as these compounds facilitate fatty acid transport into mitochondria via carnitine-acylcarnitine translocase. Decreased serum acylcarnitines further indicate altered fatty acid metabolism (51). Importantly, FAHFAs attenuate ectopic lipid accumulation and metabolic dysfunction in non-adipose tissues (52), while lysoPI promotes anti-inflammatory responses through M2 macrophage polarization (53). LysoPC demonstrates hepatoprotective and anti-inflammatory propertie, with diminished levels correlating to metabolic disease progression (8). Though PI shows potential as a lipid biomarker for plasma, its physiological functions remain insufficiently characterized.

The complex gastrointestinal ecosystem presents challenges in elucidating microbial mechanisms (54). Functional interpretations of *Family_XIII_AD3011_group* and *Ruminococcus* remain context-dependent, as evidenced by their divergent roles across comparison groups in this study. While *Family_XIII_AD3011_group* demonstrates toxin-producing capabilities in disease models, it simultaneously exhibits probiotic functions in weaned piglets (55). Similarly, *Ruminococcus* contributes to SCFA production (56) yet correlates with colitis severity (57). Notably, maternal soybean oil and leucine supplementation appears to modulate inflammation through multispecies shifts. This effect may counteract genera linked to chronic inflammation in monogastrics (*Catenisphaera, Ruminococcus*) (58), malnutrition-associated microbiota disruption (*Terrisporobacter*) (59), and stress-induced dysbiosis (*Peptococcus*) (60). The significant reduction of these detrimental genera in lactating sows suggests improved inflammatory status and stress resilience.

PCL group showed slight cellular swelling, hepatocellular edema was often accompanied by elevated aminotransferases including ALT and AST levels (61). The observed macrovesicular steatosis in PHSO piglets characterized by enhanced triglyceride synthesis and hepatocellular accumulation without significant biochemical perturbations. And short-term mild degeneration of the liver does not affect metabolic function (62). Notably, combined soybean oil-leucine supplementation reversed these histological abnormalities in HSOL progeny. Elevated thymic indices across treatment groups indicate improved immune competence (63), potentially mediated by altered expression of hepatic inflammation-related genes.

Hepatic lipid metabolism is critical for the control of systemic lipid homeostasis (64). Lipid metabolism in sows and progeny was modulated by soybean oil-leucine interventions through PPAR signaling pathways. Although hepatic sampling limitations preclude direct sow liver analysis, piglet hepatic profiles may represent surrogate indicators. Crucially, PPAR pathway enrichment occurred across all comparison groups, with PPARA serving as the central regulator governing lipid uptake, transport, and β-oxidation (65), may serve as a bridge linking other lipid metabolism genes (66). The genes *ABCA1, FABP5, SLC27A2, PPARA*, and *PPARD* emerge as pivotal players intersecting metabolic syndrome, and lipid metabolism pathways. *ABCA1* expression can be upregulated through the PPAR-LXRα-ABCA1signaling axis, which is crucial for cholesterol and phospholipid efflux and atherosclerosis protection (67). Overexpression of *FABP5* is associated with enhanced PPAR signaling pathway activity and is a promising drug target (68). *SLC27A2* mediates fatty acid uptake and β-oxidation through non-gene cross-regulation of PPARs pathways (69). In addition, *RBP4* and *FABP5* also showed significant changes in the intervention of metabolic syndrome (70).

Daily rhythms in biochemistry, physiology, and behavior ultimately derive from the expression of rhythmic genes in each cell (71). Lipid factors can regulate circadian appetite and energy metabolic rhythms in the brain. The central clock regulates feeding rhythms by controlling the secretion of hormones related to feeding (72). The expression of genes that regulate piglet liver rhythms may eventually influence feed intake during pig fattening.

The expression of *IL1RAP* correlates with established inflammatory markers. Research demonstrates that antibodies targeting *IL1RAP* can reduce the expression of inflammatory cytokines and adhesion markers in endothelial cells, suggesting its potential as a future therapeutic target (73). *KLF9* is implicated in mediating cellular ischemic injury. This occurs through enhanced oxidative stress, specifically via KLF9's suppression of the antioxidant enzyme Thioredoxin Reductase 2 (*Txnrd2*), leading to increased reactive oxygen species (ROS) generation (74) and regulates glycolytic and oxidative phosphorylation pathways to maintain cellular metabolic homeostasis (75). *NR1D1*enhances macrophage defenses by modulating autophagy and lysosome biogenesis, underscoring its critical function in immune regulation (76). In the liver, *LGR4* establishes a link between peripheral circadian rhythms and lipid metabolism. *LGR4* expression exhibits a circadian pattern, and its ablation disrupts the diurnal rhythm of triglycerides, indicating its essential role in regulating the circadian oscillation of plasma lipids (77). The interactions and regulatory mechanisms of these genes delineate their importance across diverse biological processes-spanning oxidative stress, metabolic adaptation, and circadian rhythm control. Future research should prioritize optimizing inclusion levels, evaluating the long-term growth performance of offspring and elucidating the mechanisms underlying lipid-insulin crosstalk.

## 5 Conclusion

Dietary intervention with soybean oil and/or leucine during late gestation and lactation demonstrated overall positive effects on both sows and their offspring. Specifically, this study demonstrates that maternal soybean oil supplementation improves insulin sensitivity in lactating sows. Crucially, combining soybean oil with leucine yields synergistic benefits for offspring, enhancing piglet growth and modulating lipid metabolism, immunity, and circadian rhythm regulation.

## Data Availability

The datasets presented in this study can be found in online repositories: NCBI Sequence Read Archive (SRA) under BioProject accession numbers PRJNA1443619 (16S rDNA data) and PRJNA1444546 (Transcriptome data).
