# Supplementary material for: Soybean oil and leucine in late gestation and lactation improve maternal and offspring lipid metabolism and insulin sensitivity: insights from pig model
Source: Front Vet Sci. 2026 Apr 10;13:1793347. doi: 10.3389/fvets.2026.1793347 (PMC13105881; doi:10.3389/fvets.2026.1793347)
Supplement: Supplementary file 1 [file Data_Sheet_1.pdf]

Supplementary Material

Table S1. Ingredient composition of the lactation diets fed to sows from day 107 of gestation to day 21 of lactation (as-fed basis, %).

Item	C	CL	HSO	HSOL
Ingredients				
Corn	64.60	64.60	59.20	59.20
Wheat bran	12.00	12.00	12.00	12.00
Soybean meal (43%)	18.50	18.50	19.60	19.60
Soybean oil	0.90	0.90	5.20	5.20
$CaHPO_4$	0.90	0.90	0.90	0.90
limestone	1.20	1.20	1.20	1.20
Sodium chloride	0.40	0.40	0.40	0.40
Chloride choline (50%)	0.15	0.15	0.15	0.15
L-lysine-HCL (98%)	0.35	0.35	0.35	0.35
Premix[1]	1.00	1.00	1.00	1.00
L-leucine(99.95%)	-	1.00	-	1.00
Nutritional levels[2]				
Digestible energy, Mcal/kg	3.17	3.17	3.40	3.40
Crude protein	16.13	16.17	16.30	16.44
Crude fat	5.27	5.14	10.67	10.97
Calcium	0.81	0.81	0.78	0.79
Total phosphorus	0.60	0.62	0.63	0.58
Available phosphorus	0.31	0.31	0.30	0.30
SID-lysine	1.00	1.00	1.02	1.02
SID-methionine	0.25	0.25	0.25	0.25
SID-methionine +cystine	0.53	0.53	0.53	0.53
SID-threonine	0.57	0.57	0.57	0.57
SID-tryptophan	0.18	0.18	0.18	0.18
SID-valine	0.72	0.72	0.72	0.72
SID-isoleucine	1.34	1.34	1.32	1.32
SID-leucine	0.59	1.59	0.60	1.60

Note: [1] Provided per kilogram of diet: 22 mg of Cu, 154 mg of Fe, 100 mg of Zn, 50 mg of Mn, 0.56 mg of I, 0.56 mg of Co, and 0.5 mg of Se,5,000 IU of vitamin A, 500 IU of vitamin D3, 15 mg of vitamin E, 1.2 mg of vitamin K3, 1 mg of vitamin B1, 5 mg of vitamin B2, 2.5 mg of vitamin B6, 0.01 mg of vitamin B12, 10 mg of niacin, 4 mg of calcium pantothenate, 1.12 mg of folic acid, and 0.24 mg of biotin.

[2] The digestible energy, SID amino acids, and available phosphorus are calculated values, and all other nutritional levels are measured values. The results are shown as the mean values of 3 replicates.

Table S2. Primer sequences used for qRT-PCR amplification.

**Genes**	**Primer sequence (5′–3′)**
β-actin	F: AATCCTGCGGCATCCACGAAAC<br>R: GTGTTGGCGTAGAGGTCCTTGC
SLC27A2	F: AGGACGAGACGCTCACCTATGC<br>R: CCAGCCAGAGCCACACATAAGC
FABP4	F: GGTGCAGAAGTGGGATGGAAAGAC<br>R: CTGGTAGCCGTGACACCTTTCATG
FABP5	F: TCAAGAATGGGACGGGAAGGAGAG<br>R: CCCGAGTGCAGGTGACATTGTTC
PPARA	F: TGGCTTACGGCAATGGCTTCATC<br>R: GCCACAAAGAGGGAAAGGTCACTG
PPARD	F: GACAAGGCATCAGGCTTCCACTAC<br>R: TCCGCTCACACTTCTCGTACTCC
CYP8B1	F: GCAGGCAAGAAGATCCACCACTAC<br>R: TGACCATGAGCAGCACAAAGAGC
CYP7A1	F: AAGTCGCTCCTCGCTGTCCTC<br>R: GCTTCAGGGCTCCTAATCGTTTGG
CREB1	F: CACCTGCCATCACCACTGTAACG<br>R: GAATTGCTCCTCCCTGGGTAATGG
LGR4	F: GTGAAGCCATCCGAGGACTGAG<br>R: ATCCAGCCACAGATGCCGTAAC
GNG11	F: TCAAGTTGCAGAGACAACAGGTATC<br>R: TCACCAGAGGATCTTCTCCAGAAC
PER2	F: GTGACGACAGCGGCAAGGAG<br>R: TGGACGGATTGTGTTCGGACTTC
NOCT	F: TCAACGCAGAGCCAACAGAGG<br>R: TCGGCACTCAGCAGCTTGTAG
IL10RA	F: ACAACGGCATCATCCTGGGAAC<br>R: CGGAAGTCGTGGAAGATGGTCTC
IL-17B	F: TGTGTCTGGGCTGTGTGAACC<br>R: CACGGGCACCTGGCTGAAC
IL1RAP	F: ACGCCTCCTGGTTGTTCTAAGTC<br>R: TTCACCTTCGTCTCCTTCACAGC
GNA13	F: GAGCGGCAAGTCCACCTTCC<br>R: CATCCACCAGCACCCTCATACC
ABCA1	F: CAACCAGGCAGTCCAGACCATC<br>R: CCCAGAACTTCCGCTCATCCAG
DDX58	F: CACAACACCAGCAAACAGCATCC<br>R: AACCGAGGCAGTCAGTCCAATG
CD74	F: CTGCGTCAGTGGCTCTTGTTTG<br>R: ACAGGTCCTCCGTCTCCAGTG
KLF9	F: GCGGCTGCGACTACCTGAG<br>R: AAGCTCTTGGCGATGGTGACC
KNG1	F: AGGCTGTGGACACGGCTTTG<br>R: ACAGTCGCCCTCCTTGATTTCG
NR1D1	F: GTCCTCCTCCTCCTCCTCCTTC<br>R: GTGATGTTGCTGGTGCTCTTGC

DGAT2	F: GCAGTGGGTCCTGTCTTTCCTC<br>R: AACGCCAGCCAAGTGAAGTAGAG
FADS2	F: AGCCAGGATCGCACCAAGAAC<br>R: GCCAGGAGGAGGAAGAAGAACAG
RBP4	F: AGGACCCTGCCAAGTTCAAGATG<br>R: GCGTAGGTGTCATAGTCCGTGTC

Table S3. Effects of soybean oil and leucine supplementation on production and reproductive performance of sows during late gestation and lactation.

item	treatment				SEM	*P*-value		
	C	CL	HSO	HSOL		O	L	O×L
BW, kg								
G107 d	229.47	222.60	224.39	227.02	2.200	0.940	0.632	0.284
L7 d	203.79[a]	204.00[a]	205.22[a]	183.11[b]	1.967	0.016	0.007	0.006
L21 d	206.79[a]	212.52[a]	215.59[a]	196.02[b]	1.867	0.307	0.068	0.001
BW change（G107 d - L7 d）	-23.04[b]	-17.96[b]	-8.66[a]	-38.60[c]	1.636	0.343	<0.001	<0.001
BW change（L7 d - L21 d）	4.65	10.17	6.58	12.27	1.557	0.519	0.077	0.979
ADFI, kg								
G107 d - G113 d	2.56	2.56	2.57	2.56	0.460	0.535	0.595	0.568
Lactation Week 1	2.78	2.90	2.61	2.58	0.632	0.089	0.734	0.562
Lactation Week 2	5.49	5.49	5.50	5.50	0.223	0.800	0.988	0.988
Lactation Week 3	6.74	6.85	6.85	6.86	0.230	0.228	0.228	0.309
L1 d - L21 d	15.01	15.21	14.97	14.94	0.619	0.246	0.512	0.380
BF, mm								
G107 d	17.72	17.46	16.47	16.71	0.404	0.219	0.989	0.759
L7 d	14.67	14.20	14.46	14.99	0.614	0.954	0.390	0.288
L21 d	19.39[b]	19.84[b]	22.99[a]	20.24[b]	0.413	0.018	0.170	0.057
BF change（G107 d - L7 d）	-2.97	-3.16	-3.56	-3.59	0.246	0.306	0.826	0.877
BF change（L7 d - L21 d）	4.27[c]	6.38[b]	9.27[a]	6.73[b]	0.428	<0.001	0.762	0.002
number of healthy piglets	12.12	12.35	12.65	12.65	0.284	0.471	0.837	0.837
number of stillbirths	1.47	1.29	1.41	1.18	0.125	0.639	0.349	0.814
estrus rate over 7 days post-weaning, %	70.00	75.00	75.00	78.57	0.074	0.773	0.773	0.962

Note: Data were presented as means with SEM (n = 17). [a-c] Different shoulder markers represent significant differences ($P < 0.05$).

Table S4. Effects of supplementation of soybean oil and leucine in sow diets on plasma biochemical parameters of piglets.

Item	Treatment				SEM	*P*-value		
	PC	PCL	PHSO	PHSOL		O	L	O×L
Piglets								
TG (mmol/L)	0.74[ab]	0.49[b]	0.68[ab]	0.88[a]	0.049	0.114	0.846	0.033
TC (mmol/L)	14.19	12.75	10.89	10.52	1.388	0.330	0.749	0.849
LDL (mmol/L)	10.54	11.46	10.14	10.00	1.308	0.726	0.883	0.842
HDL (mmol/L)	3.02	1.87	1.32	2.71	0.332	0.523	0.864	0.070
ALT(U/L)	12.97	15.10	18.37	10.04	1.454	0.954	0.303	0.091
AST(U/L)	20.75	27.82	22.53	30.44	2.321	0.643	0.126	0.930
GLU (mmol/L)	4.59	4.46	4.70	3.74	0.286	0.600	0.352	0.478
GSP (mmol/L)	1.18	1.19	1.17	1.12	0.033	0.586	0.776	0.644
INS (mIU/L)	29.52	30.06	29.84	31.55	0.618	0.475	0.373	0.642
FFA(μmol/mL)	1.32[ab]	1.20[ab]	0.76[b]	1.91[a]	0.094	0.767	0.048	0.018
TBA(μmol/L)	12.37	12.45	14.01	14.83	1.503	0.512	0.881	0.904
CHE(U/L)	48.34	30.80	48.22	45.03	6.653	0.603	0.447	0.597
γ-GT(U/L)	65.91	51.66	60.20	56.74	3.829	0.968	0.263	0.490
BUN (mmol/L)	4.18	3.53	2.97	3.38	0.373	0.374	0.872	0.484
UA(μmol/L)	45.27	35.95	48.81	42.27	3.034	0.429	0.210	0.821
FGF21(ng/L)	203.88[a]	169.36[b]	186.04[ab]	174.17[ab]	5.255	0.542	0.039	0.294

Note: Data were presented as means with SEM (n = 6). [a-c] Different shoulder markers represent significant differences ($P < 0.05$).

Figure S1.

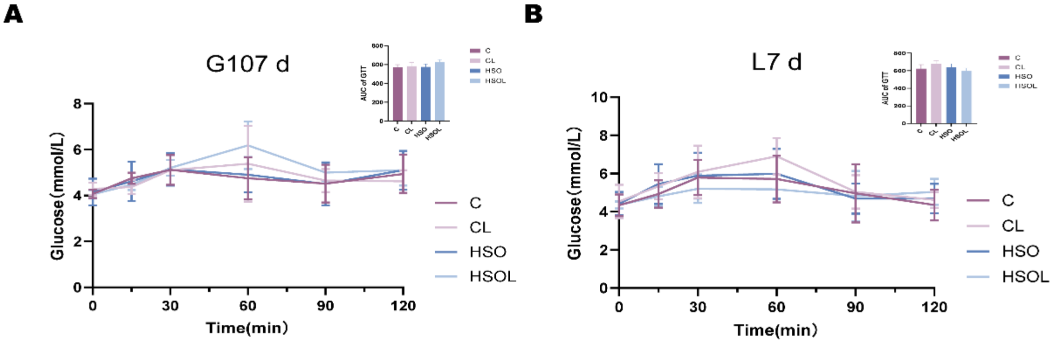

Figure S1. Sow glucose tolerance test (GTT). Glucose tolerance test (GTT) was performed on sows in each group on day 107 of gestation (A) and day 7 of lactation(B) (n = 6). Data are means ± SEM.

Figure S2.

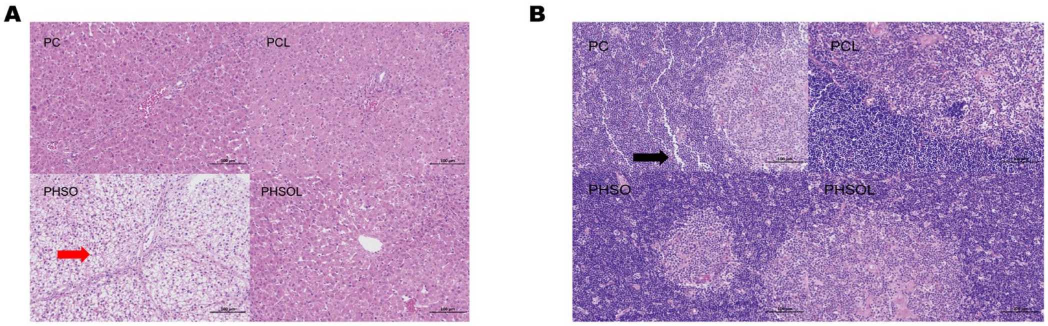

Figure S2. Liver and thymus histological morphology of piglets. (A) HE staining of piglet liver (magnification 200×), macro vesicular steatosis was observed (red arrow) (n=3). (B) HE staining of piglet thymus (magnification 200×), an enlarged gap (black arrow) around the cortical and medullary union (n=3).
